# Comparison of electromagnetic navigation bronchoscopic localization and computed tomographic imaging-guided methylene blue localization in the resection of pulmonary nodules: a retrospective cohort study

**DOI:** 10.7717/peerj.19224

**Published:** 2025-04-04

**Authors:** Babou Sowe, Junhao Wan, Fengjing Yang, Chuangyan Wu, Sihua Wang, Song Tong

**Affiliations:** Department of Thoracic Surgery, Union Hospital Affiliated to Tongji Medical College, Huazhong University of Science and Technology, Wuhan, Hubei, China

**Keywords:** Pulmonary nodule, Electromagnetic navigation bronchoscopy, Computed tomographic imaging-guided localization, Methylene blue, Video-assisted thoracoscopic surgery (VATS)

## Abstract

**Background:**

Accurate localization of small-to-medium pulmonary nodules before video-assisted thoracoscopic surgery (VATS) is essential for success. Traditional imaging methods face challenges in the thoracic cavity. This study compares electromagnetic navigational bronchoscopic (ENB), computed tomography (CT)-guided localization effectiveness and safety with methylene blue marker dye.

**Materials and Methods:**

This study, approved on May 4, 2024 by the Institutional Review Board of Wuhan Union Hospital (IRB ID UHCT240340) affiliated with Tongji Medical College, included patients with pulmonary nodules scheduled for VATS. Methylene blue was used as a marker dye and injected via CT-guided percutaneous or ENB techniques. The study compared clinical parameters, success rates, and complications between the two localization methods.

**Results:**

Out of 378 patients who underwent preoperative localization, 254 received electromagnetic navigational bronchoscopy (ENB) and 124 had CT-guided percutaneous marker dye injections. Nodules were significantly larger in the ENB group (*p* < 0.001). Success rates were similar: ENB at 97.24% and CT-guided at 97.58%. ENB was more effective in the upper lobes than the lower lobes (*p* = 0.005), with no lobar preference for CT-guided localization (*p* = 0.073). ENB also had significantly fewer complications than CT-guided procedures (*p* < 0.001).

**Conclusions:**

ENB localization success rates are comparable to CT-guided methods, but ENB carries lower risks, particularly for puncture-related complications. CT-guided localization is more effective than ENB for lower lobe nodules.

## Introduction

Lung cancer remains the most prevalent type of cancer and one of the most frequent causes of cancer death in men and women worldwide (Bade & De la Cruz, 2020). Most patients reach an advanced stage at diagnosis, which in most cases leads to poor prognosis or death. The five-year survival rate for patients with stage I disease who undergo radical resection is reported to be between 68% and 92% (Yang et al., 2021).

Detecting lung cancer early is crucial for effectively managing the disease. Surgical removal is widely considered the best treatment for early-stage lung cancer. As lung cancer screening methods like low-dose computed tomography (CT) become more widely available and accessible, the incidence of pulmonary nodules is increasing. These screening techniques allow for the quick detection and removal of identified pulmonary carcinomas, which promotes early diagnosis and treatment.

Despite the swift invention of technological methods that ease the localization of small pulmonary nodules, identifying and marking such pulmonary lesions may remain a challenge to some surgeons. Therefore, various techniques have been developed to support the localization of such lesions within the lungs. These techniques include CT imaging-guided localization *via* the injection of marker dye or hook wire insertion, electromagnetic bronchoscopic localization *via* the injection of marker dye (Xue et al., 2020; Tian et al., 2020; Mariolo et al., 2021; Xu et al., 2021; Zhu et al., 2022; Xia et al., 2024). However, there is currently no universally accepted standard for preoperative localization techniques.

Electromagnetic navigation bronchoscopy (ENB) is a recently developed localization technique that enables surgeons to reach peripheral pulmonary lesions. This innovative approach utilizes an image-guided flexible catheter and a dedicated navigation software system to facilitate precise navigation within the lungs (Xue et al., 2020; Mariolo et al., 2021; Xu et al., 2021; Yao et al., 2023; Dunn et al., 2023; Chan et al., 2023; Bondue et al., 2023; Guo et al., 2022; Lee et al., 2022; Uzbeck et al., 2022; Zhang et al., 2021; Yang et al., 2021; Jiayuan, 2021; Piao et al., 2020; Cheng & Chu, 2019; Folch et al., 2019; Hyun et al., 2019; Qian et al., 2019). After the identification of the landmark of the lesion, a marker dye (methylene blue, indocyanine green, *etc.*) is injected through the catheter to the localization point within the lungs. Then video-assisted thoracoscopic surgery (VATS) will be done to resect out the nodule.

In this study, we retrospectively compared the safety and efficiency of CT-guided percutaneous localization and electromagnetic navigation bronchoscopic localization *via* the injection of methylene blue as a marking dye to research the most accurate and safest technique for the preoperative localization of pulmonary nodules. The study assessed the success rate of dye localization by determining the proportion of patients in whom the intended lesions were accurately identified.

## Patients and Methods

### Patient demographics and study design

Patients who were enrolled for sub-lobar resection of pulmonary nodules with a maximum nodule diameter <20 mm at the Department of Thoracic Surgery of Union Hospital, Tongji Medical College, Huazhong University of Science and Technology (Wuhan, China) between October 2021 and October 2023 were included in this study. The patients were assigned to either CT-guided or ENB localization according to the location of their lesion(s) (distance of the lesion from the pleura) and the possible complications that may arise just after conducting the procedure. Data recorded from these techniques was retrieved from patient medical records, which included the age of each patient, gender, height, weight, nodule size, nodular depth, incisal margin, smoking history, family history of cancer, number of nodules, CT characteristics (ground-glass nodule, mixed ground-glass nodule, solid), operation method, lesion depth from pleura, location, and complications observed. This study was approved and the informed of consent from patients was waived on the 4th of May 2024 by the Institutional Review Board of Union Hospital (IRB ID number UHCT240340) affiliated with Tongji Medical College of Huazhong University of Science and Technology, making the data collectors fully certified to have access to the data required for the analysis. We got access to the database of our hospital, Union Hospital, on the 17th of May 2024 to collect the raw data that we need to analyze the possible outcomes of this research.

### Percutaneous CT imaging-guided localization

Depending on the location and accessibility of the lesion, the patient is placed in the appropriate position (supine, prone, or lateral). A preoperative CT scan of the chest wall was conducted utilizing a radiopaque grid. Subsequently, under local anesthesia with lidocaine, a needle containing a marking solution (0.3 mL of Methylene blue) was injected near the lung nodule. Postoperative CT imaging was performed to monitor for the occurrence of delayed hemopneumothorax.

### ENB localization, dye marking, and surgical procedure

Patients undergoing ENB with bronchial localization of pulmonary nodules were identified at a preoperative planning CT scan performed before every ENB procedure. In the planning stage, standard format thin-slice CT scan data was imported into the ENB system (LungCare navigation system; LungCare Medical Technologies, Suzhou, China) to reconstruct the 3D virtual airway road map. Navigational bronchoscopy is performed by experienced interventional pulmonologists using the ENB system. Upon approaching the target area with the fluoroscope, 0.3 mL of methylene blue dye was administered. Based on the specific requirements, the anesthesiologist then proceeded with one-lung ventilation using a dual-lumen endotracheal tube. The surgical procedures performed through video-assisted thoracoscopic surgery (VATS) included wedge resection, segmentectomy, or both. Following removal, the tissue sample was sent for histopathological analysis to ascertain the nature of the lung nodule(s).

### Statistical analysis

Technical success is defined as dyes within lesions, or although dyes are not within lesions, the operator can successfully find the nodule through the relative positional relationship between dyes and lesions, and it is resected with an adequate margin. Continuous variables are expressed as mean ± standard deviation (S.D.), and categorical variables are expressed as numbers (%). *χ*2 test and *t*-test were used to determine whether the proportion and mean of the two groups were statistically different. The clinical characteristics of different groups were compared by single-factor analysis. All the analyses were performed with EmpowerStats (http://www.empowerstats.com; X&Y Solutions, Inc., Boston, MA, USA) and the statistical software package R (http://www.R-project.org; The R Foundation). The confidence interval was 95%. A *P*-value of < 0.05 (two-sided) was considered statistically significant.

## Results

### Characteristics of the patients

A total of 378 patients, including 254 and 124 patients underwent preoperative localization by ENB and CT-guided percutaneous marker dye injection respectively. No patient underwent all the two procedures (CT-guided and ENB) simultaneously before undergoing VATS. The clinical parameters of the patients and pulmonary nodules are summarized in Table 1. The maximum nodule diameter of CT-guided localization was smaller than that of ENB localization (*P* < 0.001). The nodule depth is similar in the two groups (*p* = 0.266). Nodule density was different in the two groups (*p* = 0.021), and pure ground-glass opacity (GGO) seemed to account for more in the CT-guided localization group (35.48%) than in the ENB group (25.98%). The proportion of bronchus signs in the ENB group was higher than in the CT-guided localization group (*p* = 0.021). Operative methods were similar in both groups (*p* = 0.463).

**Table 1:** Characteristics of the study population.

	ENB	CT-guided localization	*P*-value
No. of patients	254	124	
Age	53.48 ± 11.51	50.63 ± 10.93	0.022
Height	163.44 ± 7.26	163.93 ± 7.33	0.539
Weight	62.03 ± 10.97	62.08 ± 10.67	0.967
Maximum nodule diameter	10.08 ± 4.70	8.25 ± 3.68	<0.001
Nodule depth	7.61 ± 8.61	8.65 ± 8.25	0.266
Incisal margin	19.76 ± 9.18	20.54 ± 11.32	0.476
Gender			0.761
Male	76 (29.92%)	39 (31.45%)	
Female	178 (70.08%)	85 (68.55%)	
Smoke			0.076
Never smoking	229 (90.16%)	109 (87.90%)	
Smoked previously	21 (8.27%)	8 (6.45%)	
Smoking	4 (1.57%)	7 (5.65%)	
Family history of cancer			0.197
No	215 (84.65%)	111 (89.52%)	
Yes	39 (15.35%)	13 (10.48%)	
No. of Nodules			0.651
1	215 (84.65%)	100 (80.65%)	
2	33 (12.99%)	21 (16.94%)	
3	5 (1.97%)	3 (2.42%)	
4	1 (0.39%)	0 (0.00%)	
Nodule density			0.021
Pure GGO	66 (25.98%)	44 (35.48%)	
Mixed GGO	159 (62.60%)	75 (60.48%)	
Solid	29 (11.42%)	5 (4.03%)	
Bronchus sign			0.021
No	214 (84.25%)	115 (92.74%)	
Yes	40 (15.75%)	9 (7.26%)	
Operative methods			0.463
Wedge resection	131 (51.57%)	63 (50.81%)	
Segmentectomy	120 (47.24%)	61 (49.19%)	
Wedge resection+ segmentectomy	3 (1.18%)	0 (0.00%)	
Location Success			0.848
Yes	247 (97.24%)	121 (97.58%)	
No	7 (2.76%)	3 (2.42%)	

**Notes.**

GGO Ground Glass Opacity

### Location results of nodules

The overall success rate of each technique is as follows: ENB localization was 247/254 (97.24%) and that of CT-guided localization was 121/124 (97.58%) (Table 1). In subgroup analysis, we found that the lower lobe localization success rate decreased significantly compared to the upper lobe in the ENB group (*p* = 0.005), and there was a similar trend in the CT-guided localization group, but the statistical difference was insignificant (*p* = 0.073). Subgroup analysis of distance from pleura, nodule size, nodule density, and bronchus sign showed no significant difference in the localization success rate (Table 2). The localization complications in the CT-guided localization group were significantly higher than in the ENB group (*p* < 0.001) (Table 3). In the CT-guided localization group, 9 out of 124 patients (7.3%) showed complications: 6 out of 121 (5.2%) had hemopneumothorax and 3 out of 121 (2.6%) had hemoptysis. However, no complications were observed in the ENB localization group, with 0 out of 254 patients (0.0%) showing hemopneumothorax or hemoptysis.

**Table 2:** Location results in different groups.

	ENB		*P*-value	CT-guided localization		*P*-value
	Success	Fail		Success	Fail
Distance from pleura			0.111			0.218
<=10	175 (70.85%)	3 (42.86%)		80 (66.12%)	3 (100.00%)	
>10	72 (29.15%)	4 (57.14%)		41 (33.88%)	0 (0.00%)	
Nodule size, mm			0.711			0.442
<=10	158 (63.97%)	4 (57.14%)		101 (83.47%)	3 (100.00%)	
>10	89 (36.03%)	3 (42.86%)		20 (16.53%)	0 (0.00%)	
Lobe location			0.005			0.073
Right upper	88 (35.63%)	0 (0.00%)		48 (40.68%)	0 (0.00%)	
Right lower	49 (19.84%)	3 (42.86%)		26 (22.03%)	2 (66.67%)	
Left upper	70 (28.34%)	0 (0.00%)		35 (29.66%)	0 (0.00%)	
Left lower	40 (16.19%)	4 (57.14%)		9 (7.63%)	1 (33.33%)	
Nodule density			0.771			0.929
Pure GGO	65 (26.32%)	1 (14.29%)		43 (35.54%)	1 (33.33%)	
Mixed GGO	154 (62.35%)	5 (71.43%)		73 (60.33%)	2 (66.67%)	
Solid	28 (11.34%)	1 (14.29%)		5 (4.13%)	0 (0.00%)	
Bronchus sign			0.345			0.624
No	209 (84.62%)	5 (71.43%)		112 (92.56%)	3 (100.00%)	
Yes	38 (15.38%)	2 (28.57%)		9 (7.44%)	0 (0.00%)	

**Table 3:** Characteristics of the study population.

	ENB	CT-guided localization	*P*-value
Complications			<0.001
No complications	254 (100.0%)	115 (92.7%)	
With complications	0 (0.0%)	9 (7.3%)	
Hemopneumothorax	0 (0.0%)	6 (5.2%)	
Hemoptysis	0 (0.0%)	3 (2.6%)	

## Discussions

In this study, we realized very high success rates in using percutaneous CT-guided or ENB *via* dye marking for the localization of peripheral lesions with subsequent VATS resection. We utilized methylene blue as a marker dye because of its ease of convergence around the target lesion and visibility to surgeons when the thoracoscope is inserted in the patient’s thorax. These led to the high feasibility of all the two localization techniques and showed their comparability. However, CT-guided, which is an invasive technique, may require multiple insertion points when there are numerous nodules located in different lobes or located bilaterally in the two lungs of a person. Compared to the CT-guided technique, the ENB marking procedure has shown much more safety than other techniques in patients with multiple nodules, because it is less invasive and time-saving (Bondue et al., 2023; Guo et al., 2022; Fu et al., 2023; Tsai et al., 2023).

Secondly, in comparing our results with the existing literature, we found that our success rates for ENB (97.24%) and CT-guided localization (97.58%) are consistent with those reported in prior studies. For instance, Folch et al. (2019) and Khandhar et al. (2017) reported ENB success rates of 88–95%. Our observed complication rates also align with prior findings, with ENB demonstrating significantly fewer complications (0%) compared to CT-guided localization (7.3%). This is consistent with studies reporting higher rates of pneumothorax (5–15%) and hemorrhage (1–5%) for CT-guided procedures (Khandhar et al., 2017; Folch et al., 2019). This consistency of our results with existing literature has shown the dependability of our study.

Moreover, our study showed that the maximum nodule diameter of CT-guided localization was smaller than that of ENB localization, which was in contrast to the Tian et al. (2020) results, which showed that the ENB group located smaller lung nodules. Tian et al. (2020) study used hook wire instead of methylene blue in their CT-guided localization, which may have influenced their allocation of patients while a single localization marker (methylene blue) was used in our studies. This may be related to the subjective choice of the doctor who performed the localization technique, because in our hospital, CT-guided localization has been carried out for a longer time, and the doctor is more confident in locating smaller nodules than ENB. This phenomenon may disappear as the number of ENB cases increases. Our investigation suggested that the lower lobe localization failure rate increased significantly compared to the upper lobe in the ENB group, with no lobar predilection for CT-guided localization. Previous studies on ENB biopsies showed a similar trend, with their results showing higher biopsy success in the upper lobe (Bhatt et al., 2018; Makris et al., 2007). One important reason for this may be the length of the bronchoscope, that was used in the ENB procedure. Since the lower lobes are more distant in their anatomic location, bronchoscopes shorter in length may not reach them satisfactorily, which has led to a significant difference in the results as shown in our studies. This suggests that CT-guided localization has an advantage over ENB localization in the lower lobe. We recommend the extension of the length of the bronchoscopes used for ENB to address this difference. The finding that ENB is less effective for lower lobe nodules is a novel insight that warrants further investigation, therefore, a larger sample size of multicentre data may be required to confirm this suggestion.

This study also showed that both techniques have strengths and limitations. The CT-guided localization method is a well-established technique known for its high success rate, reaching as high as 97.58% (Table 2). However, complications stemming from pleural punctures, such as those encountered during multiple lesion localizations, remain a primary drawback of this approach (Yang et al., 2021). Additionally, there are concerns regarding the increased radiation exposure associated with bilateral pulmonary lesions because the practitioner will spend a longer period in the imaging room to localize bilateral nodules, which poses another limitation to its widespread use (Yang et al., 2021). Patients with emphysematous lung disease or chronic obstructive pulmonary disease (COPD) are not typically recommended to undergo CT-guided localization due to the higher risk of complications associated with this procedure. These complications may include hemopneumothorax, hemoptysis, and bronchial hemorrhage, which can pose significant risks to individuals with pre-existing lung conditions. Therefore, alternative localization techniques, such as electromagnetic navigation bronchoscopy (ENB), may be considered safer for patients with emphysematous lung disease or COPD (Theilig et al., 2020; Maalouf et al., 2022).

ENB offers several advantages, including enhanced patient comfort due to its performance under general anesthesia. Additionally, the procedure seamlessly transitions from localization to surgery without the need to adjust settings, thereby reducing procedure duration and associated risks (Xia et al., 2024; Khandhar et al., 2017; Gu et al., 2017; Muñoz-Largacha et al., 2017). The ENB enables access to areas that may be challenging to reach using the CT-guided approach. This encompasses nodules located close to structural reference points, for instance, the costophrenic angle, or underneath the scapula, covering areas like the side facing the mediastinum and the upper back region concealed by the scapula (Yang et al., 2021). In patients suffering from emphysematous lung conditions or chronic obstructive pulmonary disease (COPD), electromagnetic navigation bronchoscopy (ENB) is advisable because it has a proven safety record, which is supported by the absence of adverse events like hemopneumothorax, coughing up blood, and bleeding in the airways, as observed in this research (Table 3). However, ENB has its limitations. Due to its complexity and the requirements needed to perform it swiftly, it requires a high level of skill and experience from an endoscopist, an experienced anesthesiologist, and a highly developed network system to enable its performance ease, making it less readily available in many institutions. Additionally, ENB is a relatively new technique that is only feasible and affordable in high-grade hospitals, limiting its accessibility to a broader population. In addition, the lower lobe nodule localization failure rate of ENB localization was higher than that of CT-guided localization. As for centrally located tumors, the methylene blue disrupts the surgeon’s vision leading to peripheral resection that may require further resection (wedge+segmentectomy) (Table 1).

In addition, our study found that ENB had a significantly lower success rate for nodules in the lower lobes compared to the upper lobes (*p* = 0.005), while CT-guided localization showed no significant lobar preference (*p* = 0.073). This suggests that anatomical location plays a critical role in the effectiveness of ENB, particularly in the lower lobes, where longer and more tortuous bronchial pathways may pose challenges for navigation and dye placement. The higher success rate of ENB in the upper lobes may be attributed to the shorter and more straightforward bronchial pathways, which facilitate easier navigation and dye placement. In contrast, the lower success rate in the lower lobes highlights the limitations of ENB in accessing more distal and anatomically complex regions of the lung. CT-guided localization, which relies on direct percutaneous access, appears to be less affected by these anatomical challenges, making it a more reliable option for lower lobe nodules. These findings have important clinical implications. ENB may be preferred for upper lobe nodules, where its success rate is higher and the risk of complications is lower. Conversely, CT-guided localization may be more suitable for lower lobe nodules, particularly in cases where ENB is less effective due to anatomical constraints. Future research should explore the anatomical factors that contribute to these differences, such as bronchial branching patterns and nodule depth, to further refine the selection of localization techniques.

Finally, administering methylene blue provides a cost-effective, reliable, and safe technique for localizing structures within the chest cavity. This is due to the rapid convergence of the dye around or near the target lesion and its visibility to surgeons when using a thoracoscope. Consequently, this technique has found applications in the localization of pulmonary nodules. The study also provides real-world experience and calls for further large-sample studies comparing it directly with other techniques. These factors contribute to the reliability of our results and make our recommendations clear for evidence-based practice. While our study benefits from a diverse patient population drawn from various regions within China, the generalizability of our findings may be influenced by genetic, ethnic, and regional factors. For example, East Asian populations have been shown to have a higher prevalence of ground-glass nodules (GGNs) and distinct molecular profiles (*e.g.*, epidermal growth factor Receptor (EGFR) mutations) compared to Western populations. These differences could impact the success rates and complications of localization techniques. Future multicenter studies involving diverse populations are needed to further explore these factors and enhance the external validity of our findings.

### Study limitations

While our study provides valuable insights into the comparative effectiveness of ENB and CT-guided localization, several limitations should be acknowledged. First, the retrospective design introduces the potential for selection bias, particularly in the allocation of patients to the ENB or CT-guided groups. Although we used univariate and multivariate analyses to control for confounding variables, advanced statistical techniques such as propensity score matching (PSM) or inverse probability weighting (IPW) could further mitigate bias and enhance the validity of the findings. Future studies should consider employing these methods to ensure a more balanced comparison between the two techniques.

Secondly, the allocation of patients was not randomized and had no blinding, which may result in observer bias. To address this, our study was conducted in a real-life clinical setting, where patients were assigned to ENB or CT-guided localization based on clinical judgment, nodule characteristics (*e.g.*, size, location, depth), and potential complications. This approach reflects standard clinical practice thereby enhancing the efficacy of the performance of each technique. Given that our research was conducted in a real-life clinical setting, we recommend multicenter randomized control trial and advanced statistical techniques such as propensity score matching (PSM) or inverse probability weighting (IPW) in future studies, to create more balanced comparison groups and reduce the impact of selection bias and confirm the outcomes of our research.

Additionally, the optimal marking dye remains uncertain, leading to challenges in standardizing the dye used for pulmonary nodule localization. Therefore, factors such as location, distance from the pleura, nodule size, cost, patient characteristics, and CT findings should all be taken into account when determining the most appropriate localization technique for each patient. Our study has contributed to the literature by providing a direct comparison of ENB and CT-guided localization using a single marker dye (methylene blue) in a large patient cohort. By contextualizing our results within the broader body of research, we aim to provide a more comprehensive understanding of the strengths and limitations of these localization techniques.

Another important acknowledged limitation is that the study lacks long-term follow-up data, such as complete resection rates, recurrence rates, and survival outcomes. While the primary focus was on the technical success and immediate complications of the localization techniques because of the retrospective nature of the study, long-term outcomes are critical for evaluating the overall effectiveness and long-lasting clinical relevance of these procedures. We recommend future prospective studies to include these long-term outcomes, thereby contributing to the long-lasting efficiency of these procedures.

Moreover, another important limitation was the significant difference in nodule size between the ENB and CT-guided groups (*p* < 0.001). Nodules in the ENB group were larger, which could inherently influence the success rates and complications associated with each localization method. For example, larger nodules may be easier to localize, potentially inflating the success rate of ENB, while smaller nodules may pose greater technical challenges for CT-guided localization. To ensure a valid comparison between the two groups, it is essential to control for nodule size and other confounding factors. While we used univariate and multivariate analyses to compare outcomes, these methods do not fully account for the difference in nodule size. Advanced statistical techniques such as propensity score matching (PSM) or inverse probability weighting (IPW) could be employed in future studies to adjust for nodule size and other covariates, providing a more robust comparison of outcomes.

Finally, the absence of long-term follow-up data limits our ability to assess the durability of resection and the impact of localization techniques on patient outcomes such as recurrence rates and survival. At our institution, we are actively collecting long-term follow-up data, which will be the focus of future analyses. We recommend that prospective studies include standardized follow-up protocols to provide a more comprehensive evaluation of ENB and CT-guided localization techniques.

## Conclusions

In conclusion, the success rate of ENB localization is similar to that of CT-guided localization. Moreover, the ENB approach allows for swift localization of pulmonary nodules without imposing patient risk, particularly regarding puncture-related complications like hemopneumothorax or hemoptysis. As such, the ENB approach may be considered comparable to the CT-guided approach for identifying and localizing pulmonary nodules. The use of CT-guided localization is more advantageous than the use of ENB localization in the lower lobes.

##  Supplemental Information

10.7717/peerj.19224/supp-1 Supplemental Information 1 Raw Data All the parameters that were studied in this research.

## Abbreviations

VATS: Video-assisted thoracoscopic surgery
ENB: Electromagnetic navigation bronchoscopy
CT-guided: Computed tomographic imaging-guided localization
IRB ID No.: Institutional Review Board Identification Number
COPD: Chronic Obstructive Pulmonary Disease

## Additional Information and Declarations

### Data Availability

The following information was supplied regarding data availability: The raw data is available in the Supplemental File.
